# Modification of Non-Vector Aphid Feeding Behavior on Virus-Infected Host Plant

**DOI:** 10.1673/031.013.2801

**Published:** 2013-04-12

**Authors:** Zuqing Hu, Huiyan Zhao, Thomas Thieme

**Affiliations:** 1 State Key Laboratory of Crop Stress Biology in Arid Areas, college of plant protection, Northwest A&F University, Yangling, 712100, Shaanxi, China; 2 BTL Bio-Test Labor GmbH Sagerheide, Birkenallee 19, 18184 Sagerheide, Gemany

**Keywords:** *Sitobion avenae* (Fabricius), electrical penetration graph, *Wheat dwarf virus*, *Cereal yellow dwarf virus -* RPV, virus infection, virus-free vector pre-infestation

## Abstract

Virus-infected host plants can have positive, neutral or negative effects on vector aphids. Even though the proportion of non-vector aphids associated with a plant far exceeds that of vector species, little is known about the effect of virus-infected plants on non-vector aphids. In the present study, the English grain aphid *Sitobion avenae* (Fabricius) (Hemiptera: Aphididae), a non-vector of *Wheat dwarf virus* (WDV) and *Cereal yellow dwarf virus*-RPV (CYDV-RPV), was monitored on, virus-infected, virus-free and leafhopper/aphid-infested, and virus- and insect-free (control) barley, *Hordeum vulgare* L. (Poales: Poaceae), plants. Electrical penetration graph recordings were performed. Compared with the control plants, *S. avenae* on infected plants exhibited reduced non-probing and pathway phase, and increased phloem sap ingestion phase, and more aphids reached sustained phloem ingestion. However, the electrical penetration graph parameters described above showed no significant differences in aphid feeding behavior on virus-free and vector pre-infested plants and the control barley plants during *S.* *avenae* feeding. The results suggest that WDV/CYDV-RPV-infected host plants positively affected the feeding behavior of the non-vector aphid *S. avenae.* Based on these results, the reasons and trends among the virus-infected host plants' effects on the feeding behavior of non-vector aphids are discussed.

## Introduction

*Wheat dwarf virus* (WDV) (Geminiviridae: Mastrevirus) is mainly transmitted by the leafhopper *Psammotettix alienus* (Dahlbom) in Europe (Schubert et al. 2007), and *Cereal yellow dwarf virus*-RPV (CYDV-RPV) (Luteoviridae: Luteovirus) is transmitted specifically by the aphids *Rhopalosiphum padi* and *Schizaphis graminum* (Stern and Vernon 1967; Irwin and Thresh 1990). The diseases caused by WDV and *Barley yellow dwarf virus* (BYDV), including CYDV, have been recognized as two of the most serious viral diseases of crops (Jiménez-Martínez et al. 2004), threatening barley and wheat production and causing significant economic losses throughout the world (Bishop and Sandvol 1984; Huth 2000; Wu et al. 2002; Ramsell et al. 2008).

Virus infection causes both physiological and biochemical changes in host plants (Jensen et al. 1971). Compared with non-infected plants, the total free amino acids were increased 150 to 180% in *Tomato spotted wilt tospovirus-*infected tomato leaves (Selman et al. 1961). Spring wheat plants infected with BYDV had altered amino acid composition compared with healthy plants. BYDV infection increased the total amino acid content of the sampled wheat leaves at different plant developmental stages, and more so at the later stages (Ajayi 1986). In addition, the chlorophyll content and the rate of photosynthesis were reduced in BYDV-infected wheat leaves (Jensen and Sambeek 1972).

Virus-induced changes in plant metabolism can influence insects, including phloem-feeding aphids. Plants infected with phytoviruses have been reported to affect vector-aphid feeding behavior and physiometry, with effects ranging from positive through neutral to negative (Eigenbrode et al. 2002; Srinivasan and Alvarez 2007; Fereres and Moreno 2009). The feeding behavior (particularly superficial tissue probing and sustained phloem sap ingestion) of the vector *Myzus persicae* could be enhanced on plants infected by the *Potato leaf roll virus* (Alvarez et al. 2007). In contrast to the positive effect, the vector *Sitobion avenae* (Fabricius) (Hemiptera: Aphididae) had similar feeding behavior parameters on BYDV-PAV-infected and non-infected wheat plants, such as time to committed phloem ingestion or total ingestion time (Fereres et al. 1990b). Moreover, *Bean yellow mosaic virus*-infected beans were reported to negatively affect settling and the performance of *Acyrthosiphon pisum* (Power 1996). *Aphis glycines* density in the field and population growth rate in laboratory assays were significantly lower on virus-infected soybean plants compared with uninfected control (Donaldson and Gratton 2007).

Previous studies have focused on the effects of virus-infected plants on vector-insect biology. However, under field conditions, most insect herbivores do not serve as vectors for plant pathogens. The effect of virus-infected plants on non-vectors has not yet been reported, even though it is fundamental to understanding non-vector-insect population in the virus-infected field and for the development of sound pest management strategies in diverse and complex agro-ecosystems. Therefore, the present study employed a designed a series of experiments to examine the feeding behavior of the non-vector aphid *S. avenae* on WDV/CYDV-RPV-infected barley plants. WDV is transmitted by leafhoppers, and CYDV-RPV is specifically transmitted by the aphids *R. padi* and *S. graminum.* The electrical penetration graph (EPG) technique was frequently employed to characterize a hostplant effect on sap-feeding insects, as it allows explaining interactions between insects' feeding behaviors and plant tissues (McLean and Kinsey 1967; Tjallingii 1988, 2006). Using the DC-EPG technique, feeding activities of aphids occuring before and during sap ingestion from phloem sieve elements were analyzed. According to the cascade effect of phytoviruses on aphids through the plant, it was hypothesized that virus-infected plants could modify the feeding behavior of non-vector-aphids, as previously reported for vector aphids. The objectives of this study were to demonstrate various changes in the feeding behavior of *S. avenae* on the WDV/CYDV-RPV-infected, WDV/CYDV-RPV-free and vector pre-infested, and insect- and virus-free (control) barley plants. The results of the study will be helpful in understanding the role of WDV/CYDV-RPV in interactions between barley and *S. avenae.*

## Methods and Materials

### Culture of plants

The experiments were conducted in environmental growth chambers located at the Bio-Test laboratory, Sagerheide, Germany. Seeds of barley, *Hordeum vulgare* L. (Poales: Poaceae) (variety ‘Lomerit,’ Intergrano Agrohandel Sp. z o.o. Lubuskie, Poland), were cultivated individually in plastic pots (14 cm in height, 12 cm in diameter) with growing medium (N:P:K = 20:20:20, Einheitserde-und Humuswerke Gebr. Patzer GmbH & Co. KG, http://www.intergrano.pl/) in a growth chamber at 20 ± 1° C, 65 ± 5% RH, and with a photoperiod of 16:8 L:D. Growth chambers were equipped with “daylight” fluorescent bulbs (115V, Philips Company, www.philips.com) that provided 250 µE/cm2/sec of light intensity. Sprouted plants were watered regularly as needed. Plants at the second or third leaf stage were used for the experiments.

### Organisms

WDV and CYDV-RPV isolates were obtained from barley leaves collected in a barley field near Rostock (54° 09′ N, 12° 08′ E) and were maintained separately in laboratory through vector transmission on barley (Lomerit) plants. The leafhopper *P. alienus* was used as a vector for the transmission of WDV virus, and the aphid *R. padi* for CYDV-RPV virus. These two virus-free vectors were originally collected in an uninfected barley field near Rostock, and tested for the presence of WDV and CYDV-RPV using an enzyme-linked immunosorbent assay (ELISA, Opsys MR, Dynex Technologies, www.dynextechnologies.com). Viruliferous *P. alienus* of WDV, virus-free *P. aliens,* viruliferous *R. padi* of CYDV-RPV, and virus-free *R. padi* were separately maintained on barley for one year before the experiment under the growth chamber conditions described as above.

### Virus transmission and virus-free vector pre-infestation

The following five treatments, each replicated 35 times, were applied on the barley plants: 1) the virus- and insect-free plants (‘control’) (i.e., plants exposed to neither insect nor virus); 2) plants infested with WDV-free *P. alienus* (‘*P. alienus* pre-infested’); 3) plants infested with CYDV-RPV-free *R. padi* (‘*R. padi* pre-infested’); 4) plants infested with viruliferous *P. alienus* of WDV (‘WDV-infected’); and 5) plants infested with viruliferous *R. padi* of CYDV-RPV (‘CYDV-RPV -infected’). A total of 175 plants were used (35 plants per treatment, with one plant per pot).

Barley plants at the second or third leaf stage were first covered with transparent, plastic, tube-shaped cages (30 cm in height, 13.5 cm in diameter, and with a mesh screen cover on the top), and then 10 late instar nymphs of the vector were introduced into the cage and allowed to feed on the leaves. All the nymphs were removed after 72 hr, and the treated plants were maintained under the growth chamber described as above.

To further examine the vector-insect feeding effect on subsequent non-vector-aphid feeding, plants previously infested with virus-free vector insects also as treatments, the procedure was the same as described above, except WDV-free *P. alienus* and CYDV-RPV-free *R. padi* replaced the viruliferous vectors. The insect- and virus-free barley plants were used as the control plants.

### ELISA

ELISA was conducted for all plants individually 10 days after virus inoculation. The leaves were collected from each plant and kept in nylon bags at 4° C in a refrigerator. Virus diagnosis was done by DAS-ELISA as described by Gray et al. (1991). The antisera and conjugates were purchased from BIOREBA (www.bioreba.ch) (CYDV-RPV) and Loewe Biochemica (www.loeweinfo.com) (WDV). The negative threshold was defined as the negative mean plus 3× standard deviations. The results showed that all 35 (100%) barley plants were found negative for the control plants. In the *P. alienus* pre-infested and *R. padi* pre-infested barley plants, 34 out of 35 (97%) and 33 out of 35 (94%) tested negative; 34 out of 35 (97%) and 32 out of 35 (91%) of WDV-infected and CYDV-RPV-infected barley plants respectively were found positive. Thirty appropriate plants from each treatment were then selected for the following experiments.

### Aphid stock

The single apterous English grain aphids, *S. avenae,* were originally collected from a field near Rostock (54° 09′ N, 12° 08′ E), Germany, in 2008, and transferred to barley plants. The plants were maintained in insect rearing tents (60 × 60 × 10 cm, MegaView Science Co., Ltd., http://nature.bugdorm.com/)under the growth chamber conditions described as above for two years. Newly cultured barley plants were exchanged weekly. The aphids were observed every three days, and excess aphids were killed in order to keep the aphid population under a low-density condition.

### Feeding experiments

The electrical penetration graph was first introduced by McLean and Kinsey (1964) and later adapted by Tjallingii (1978). The parameters describing aphid behavior during probing and feeding are good indicators of plant suitability or interference of probing with chemical or physical factors in certain plant tissues (Mayoral et al. 1996). Using this method, one electrode is implanted into the substrate supporting the plant, and the other electrode is positioned on the dorsal region of the insect using a drop of silver stain. The circuit is completed when the insect inserts its stylet into the plant tissue in order to probe the plant to feed, and from this point on the variation in voltage can be interpreted by computer software in order to construct a penetration graph. Each waveform generated by the system characterizes a type of activity and this, together with the location of the stylet, can be related to the non-probing, pathway, and phloem phases of insect feeding (Tjallingii and Prado 2001).

The Giga-8 DC-EPG (W.F. Tjallingii, University of Wageningen, The Netherlands) was used on the abaxial face of the third fully-developed leaf from the top of an experimental plant. A thin gold wire (20 µm diameter and 2 cm long) was tethered at the dorsum of an aphid by conductive silver paint (W.F. Tjallingii), and the other electrode was inserted in the dampened soil of the potted plant. Before the aphid was used for the EPG recording, it was allowed to acclimate to the tethering by allowing the aphid to crawl on a solid surface without feeding for 1 hr. For each treatment, 30 replicates (one aphid per plant) were conducted and the recordings were conducted continuously for 8 hr during the daytime (9:00–17:00).

**Table 1.**
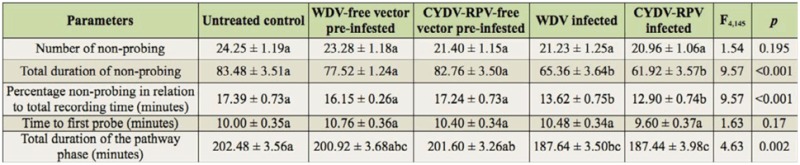
Mean ± SE values (n = 30) for non-probing and pathway phase parameters of *Sitobion avenae* on different barley treatments, respectively, obtained by the electrical penetration graph technique monitored for 8 hr. Number, total duration, and percentage of total time are indicated. Means on the same row followed by the same letter are not significantly different at *p* < 0.05. Data from each parameter were analyzed with SPSS 17.0 in separate ANOVA, followed by *t*-test pairwise comparisons. Untreated control: barley infected with neither virus nor vector; WDV: *Wheat dwarf virus*; CYDV-RPV: *Cereal yellow dwarf virus species-*RPV*.*

Aphid feeding behavior was recorded by the EPG waveforms using PROBE 3.5 software (EPG, W.F. Tjallingii). Three behavioral phases, each of which were characterized by one or more waveforms, could be distinguished: (i) non-probing phase (waveform Np), the insect is not piercing into the plant tissues; (ii) pathway phase (waveform C), forms the main activity before reaching the sieve elements in the phloem, including primary penetration through plant tissues, often with cell punctures, and salivation; and (iii) phloem phase, formed by two waveforms, E1 and E2. The E1 waveform is formed by salivation in phloem elements and the E2 waveform is formed by passive phloem sap ingestion (Tjallingii 1988). Other waveforms were acquired but not presented here because they did not provide significant information on aphid feeding behavior.

### Experimental design and Data Analysis

The current study utilized randomized complete block design. The plant status was considered as the treatment factor. The following EPG parameters were recorded and recognized through waveforms: number of non-probing events; sum of non-probing phase; time to first probe; sum of the pathway phase; number of phloem phase; sum of phloem salivation; sum of phloem ingestion; time to first phloem phase in the experiment; time to first phloem phase in probe; time to first sustained phloem ingestion in experiment; and time to first sustained phloem ingestion in probe. The data were logtransformed to fit normal distribution. The data were then separately analyzed by oneway ANOVA and a Tukey *post-hoc* test if a significant (*α* ≤ 0.05) effect was found. The percentage of aphids with phloem phase and sustained phloem ingestion phase on treatments and control plants was analyzed by χ2*-*test with continuity correction. Statistical analyses were performed using SPSS 17.0 software (www-01.ibm.com/software/analytics/spss).

**Table 2.**
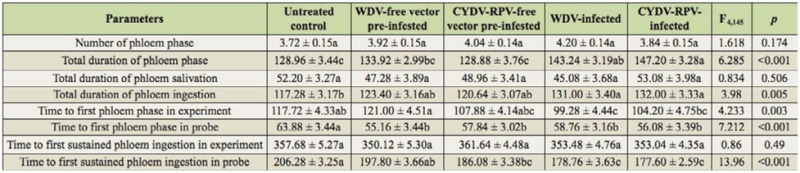
Mean ± SE values (n = 30) for the phloem phase of *Sitobion avenae* on different barley treatments, respectively, obtained by electrical penetration graph technique monitored for 8 hr. Number, total duration, and time to phase is indicated. Means on the same row followed by the same letter are not significantly different at *p* < 0.05. Data from each parameter were analyzed with SPSS 17.0 in separate ANOVA, followed by *t*-test pairwise comparisons. Untreated control: barley infected with neither virus nor vector; WDV: *Wheat dwarf virus*; CYDV-RPV: *Cereal yellow dwarf virus species*-RPV*.* All time measurements are in minutes.

## Results

### Feeding behavior of the non-probing and pathway phase

Virus infection significantly affected the sum of non-probing phase and the sum of the pathway phase (Table 1). Compared with control plants, the sum of non-probing phase and sum of the pathway phase were significantly shorter on WDV/CYDV-RPV-infected plants (Tukey *post hoc* tests: *p* = 0.001 and 0.001, *p* = 0.034 and 0.031, respectively); however they did not significantly differ from *P. alienus* or *R. padi* pre-infested plants (*p* = 0.689 and 1*, p* = 0.998 and 1, respectively). The number of non-probing events and time to first probe did not significantly differ between virus-infected or vector pre-infested plants and control plants (Table 1).

### Feeding behavior of phloem phase

Table 2 shows the non-vector aphid parameters of phloem phase on treatment and control plants. Compared with control plants, the sum of phloem ingestion was significantly larger on WDV/CYDV-RPV-infected plants (WDV-infected: *p* = 0.014; CYDV-RPV-infected: *p* = 0.026); however it was not significantly different from *P. alienus* or *R. padi* pre-infested plants (*P. alienus* pre-infested: *p* = 0.876; *R. padi* pre-infested: *p* = 0.971). The number of phloem phase and sum of phloem salivation were not significantly different among virus-infected or vector pre-infested plants compared control plants (Table 2).

Furthermore, compared with control plants, aphids fed on WDV/CYDV-RPV-infected and *P. alienus* or *R. padi* pre-infested plants showed significant shorter times for the first phloem phase (WDV-infected: *p* = 0.001; CYDV-RPV-infected: *p* = 0.006; *P. alienus* pre-infested: *p* = 0.012; *R. padi* pre-infested: *p* = 0.002). The time to first sustained phloem ingestion in probe was significantly earlier for aphids fed on WDV/CYDV-RPV-infected and *R. padi* pre-infested plants than on control plants (WDV-infected: *p* < 0.001; CYDV-RPV-infected: *p* < 0.001; *R. padi* pre-infested: *p* < 0.001). In addition, the time to first phloem phase in experiment was significantly earlier only on WDV-infected plants (*p* = 0.032). In contrast, no significant differences for the time to first sustained phloem ingestion in experiment were found between virus-infected or vector pre-infested plants compared to control plants.

### Feeding behavior in relation to the complete probing

**Figure 1.**
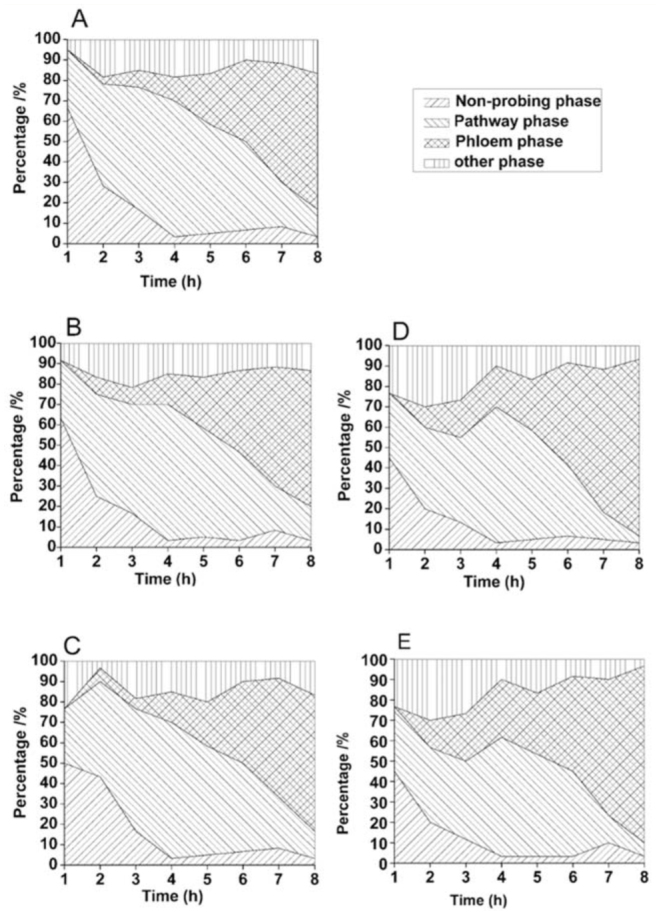
Percentage of different phases in relation to the complete probing of *Sitobion avenae* fed on different barley treatments during the 8 hr electrical penetration graph experiment. A) Control; B) *P. alienus* pre-infested; C) *R. padi* pre-infested; D) WDV-infected; E) CYDV-RPV-infected. Control: barley infected neither with virus nor vector; WDV: *Wheat dwarf virus;* CYDV-RPV: *Cereal yellow dwarf virus*-RPV. High quality figures are available online.

**Figure 2.**
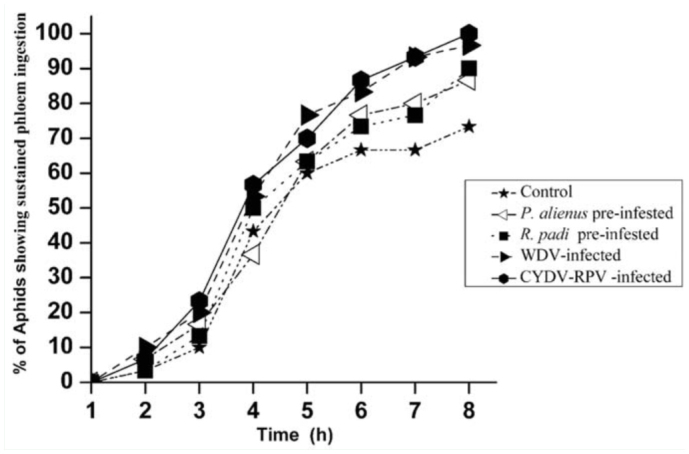
Percentage of *Sitobion avenae* reaching a sustained phloem sap ingestion on different barley treatments during the 8 hr experiment. Control: barley infected neither with virus nor vector; WDV: *Wheat dwarf virus*; CYDV-RPV: *Cereal yellow dwarf virus*-RPV*.* High quality figures are available online.

The percentage of different phases in relation to the complete probing of *S. avenae* on different barley treatments is shown in Figure 1. After 3 hr of monitoring, the proportion of *S.* *avenae* that reached phloem phase was 20.2% on WDV-infected plants and 23.5% on CYDV-RPV-infected plants, both of which were higher than on control plants (8.3%) (WDV-infected: χ2 = 5.025, *p* = 0.025; CYDV-RPV-infected: χ2 = 7.482, *p* = 0.004). At 4 hr of monitoring, the proportion was 24.6% and 26.4% on WDV/CYDV-RPV-infected plants respectively, both of which were higher than on control plants (11.6%) (WDV-infected: χ2 = 4.775, *p* = 0.029; CYDV-RPV-infected: χ2 = 5.491, *p* = 0.019). Furthermore, after 7 hr of monitoring, a higher percentage of aphids showed sustained phloem ingestion phase on the WDV/CYDV-RPV-infected plants (WDV-infected: χ^2^ = 4.007, *p* = 0.047; CYDV-RPV-infected: χ2 = 5.822, *p* = 0.016) (Figure 2). However, in the whole course of this experiment, the proportion of aphids reaching phloem and sustained ingestion phases were similar on vector pre-infested and control plants (Figure 1, 2).

## Discussion

The present study demonstrated the positive effect of WDV/CYDV-RPV-infection on *S. avenae* feeding behavior. In particular, phloem factors enhancing sieve element acceptance appear to be involved, as reflected by more aphids reaching sustained phloem ingestion within the 8 hr experiment, longer time of phloem ingestion, shorter time to first phloem phase in experiment, and shorter time to first sustained phloem ingestion in (Figure 1, 2; Table 2). On the other hand, factors from the leaf surface, epidermis, and mesophyll enhanced pathway acceptance, as reflected by the smaller sum of non-probing phase and sum of the pathway phase (Table 1). This result suggests that *S. avenae* can detect some changes in plants with viral infection during their stylet penetration towards the phloem, and these changes can be considered as an increased host plant acceptance for the aphids.

Although beneficial effects of other virus-infected plants on vector aphids have been reported (Way and Banks 1967; Way and Cammell 1970; Prado and Tjallingii 1997; Sandström et al. 2000; Gonzales et al. 2002; Sauge et al. 2002), this study showed for the first time beneficial effects of WDV (Luteovirus) or CYDV-RPV (Mastrevirus) infection on non-vector *S. avenae* feeding behavior. Our results indicate that even though an insect might not transmit a specific pathogen, feeding on the infected plant could have important consequences for its behavior, and thus significant implications for its ecology. This finding is particularly noteworthy given that for many plants, especially agricultural plants, the proportion of non-vector species associated with the crop far exceeds that of vector species. However, vector *S. avenae* had a similar feeding behavior on BYDV-PAV-infected and non-infected wheat plants (Fereres et al. 1990a, b). The results of the present study indicate *S. avenae* had different feeding behavior on transmitted virus-infected (BYDV-PAV) and non-transmitted virus-infected (WDV/CYDV-RPV) plants. Plant-mediated interactions between non-transmitted virus and *S. avenae* may fundamentally differ from interactions between transmitted virus and *S. avenae,* owing to possible competitive relationships between pathogens and *S. avenae.*

The decrease in pathway and non-probing phase, and the increased phloem sap ingestion phase, induced by virus-infected barley plants could result from the required nutrients or an enhanced host acceptance. The second hypothesis seems to fit current literature. Infection by phytoviruses has been reported to increase the amount of carbohydrates and amino acids in leaves (Markkula and Laurema 1964; Castle and Berger 1993), and the greater nutritional quality of virus-infected plants is believed to be partly responsible for improved vector life history on such plants (Fereres et al. 1989). Together with the results of the present study, such variations in diet may explain the increased development and reproduction of both green and brown clones of *S. avenae* on CYDV-RPV-infected barley plants (Hu, unpublished data).

The data from the present study also revealed, for the first time, that feeding behavior parameters for *S. avenea* exhibited no significant difference on virus-free vector-infested plants than on control plants, except the time to first phloem phase in probe (Table 2). This result suggests that previous feeding only causes *S. avenae* to reach the phloem phase earlier. The positive effect on the feeding behavior of the non-vector *S. avenae* is not related to an alleviation of the ‘anti- previous infested’ defense in WDV/CYDV-RPV-infected plants. However, the bean aphid *Aphis fabae* has been shown to not only reach phloem phase earlier, but also to have a longer phloem phase on virus-free vector-infested broad bean plants when compared to control plants (Prado and Tjallingii 1997). Further study is needed to further examine these conflicting results.

In summary, the current study demonstrates virus infections may play a role in plant— herbivore interactions. The two different viruses, CYDV-RPV (Luteovirus) and WDV (Mastrevirus), had a similar positive effect on the feeding behavior of non-vector aphid pests by increasing host plant suitability. This effect might lead to faster population increase and increased aphid damage in the virus-infected barley fields. Future studies are needed to evaluate the significance of these findings under field conditions.

##

**Abbreviations**
BYDV: *Barley* *yellow dwarf virus*
CYDV-RPV: *Cereal yellow dwarf virus*-RPV
WDV: *Wheat dwarf virus*
